# Reevaluating the Need for Routine IGF‐1 Screening in Poorly Controlled Type 2 Diabetes Mellitus

**DOI:** 10.1002/edm2.70194

**Published:** 2026-03-25

**Authors:** Munire Topalak Sonmez, Hayri Bostan, Umran Gul, Hakan Duger, Sema Hepsen, Bekir Ucan, Erman Cakal, Muhammed Kizilgul

**Affiliations:** ^1^ Department of Internal Medicine Ankara Etlik City Hospital Ankara Türkiye; ^2^ Department of Endocrinology and Metabolism Canakkale Mehmet Akif Ersoy State Hospital Canakkale Türkiye; ^3^ Department of Endocrinology and Metabolism Ankara Etlik City Hospital Ankara Türkiye; ^4^ Department of Endocrinology and Metabolism Medical Park Antalya Hospital Antalya Türkiye; ^5^ Division of Diabetes, Endocrinology, and Metabolism University of Minnesota Minneapolis Minnesota USA

**Keywords:** acromegaly, diabetes mellitus, growth hormone, IGF‐1, screening

## Abstract

**Purpose:**

Acromegaly is commonly associated with diabetes mellitus (DM), and its prevalence may be higher among individuals with type 2 DM than in the general population. This study aimed to assess the frequency of acromegaly in patients with poorly controlled type 2 DM.

**Methods:**

This cross‐sectional study included 504 patients (mean age: 57.7 ± 10.8 years) with uncontrolled type 2 DM (HbA1c > 8%) despite at least 1 year of insulin treatment (insulin alone or oral anti‐diabetic drugs together with insulin). Serum insulin‐like growth factor 1 (IGF‐1) levels were measured for screening. In patients with elevated IGF‐1, growth hormone (GH) levels were assessed by oral glucose tolerance test (OGTT). Pituitary magnetic resonance imaging (MRI) was performed in cases with persistently elevated IGF‐1 and inadequate GH suppression or discordant post‐glucose GH levels.

**Results:**

The median duration of DM was 9 (3–15) years, and the mean HbA1c was 11.12% ± 2.30%. Elevated serum IGF‐1 levels were found in 2 of the 504 patients (0.39%). Both underwent OGTT for GH evaluation. One patient showed appropriate GH suppression, and the other demonstrated borderline GH suppression (a nadir GH of 0.88 ng/mL) with a 4 × 3 mm cystic pituitary lesion on MRI. However, given the absence of acromegalic clinical features and lack of biochemical confirmation, acromegaly was not diagnosed in either case.

**Conclusion:**

Routine IGF‐1 screening in all patients with poorly controlled type 2 DM has limited diagnostic yield. IGF‐1 testing should be reserved for patients exhibiting clinical features or phenotypic suspicion of acromegaly.

## Graphical abstract

In 504 patients with poorly controlled type 2 diabetes (HbA1c > 8%), routine IGF‐1 screening identified elevated levels in only 0.39%. No patient fulfilled biochemical or clinical criteria for acromegaly after confirmatory testing. Routine IGF‐1 screening in poorly controlled diabetes has limited diagnostic yield and should be reserved for clinical or phenotypic suspicion of acromegaly.

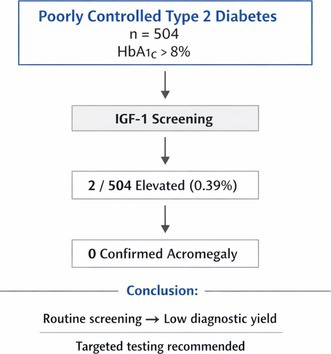

## 1 Introduction

Acromegaly is a gradually progressive disorder characterised by increased secretion of growth hormone (GH), which subsequently leads to elevated levels of insulin‐like growth factor‐1 (IGF‐1) [1]. Although rare, acromegaly has an estimated prevalence of 28–137 cases per million [2]. However, several authors suggest that these figures underestimate the true prevalence. A recent study involving 3173 patients reported that this diagnostic delay averages approximately 8–10 years [3]. Therefore, there is a critical need for new screening strategies that can improve diagnostic awareness and facilitate the earlier detection of acromegaly.

Chronic GH excess induces profound metabolic derangements—most notably insulin resistance—which contributes to impaired glucose tolerance and diabetes mellitus (DM) in approximately 15%–38% of patients [4, 5]. Conversely, studies have demonstrated a higher prevalence of acromegaly among individuals with diabetes or glucose intolerance compared with the general population, ranging from approximately 0.13% in outpatients [6] to 0.6% in hospitalised patients with type 2 DM [7]—several‐fold to over 100‐fold higher than estimates in the general population.

Although no standardised screening recommendations currently exist, clinicians—particularly endocrinologists—often consider endocrine disorders that contribute to glucose dysregulation, such as Cushing's syndrome and acromegaly, in the differential diagnosis of patients with DM. However, given the vast number of individuals affected by diabetes, the cost‐effectiveness and diagnostic yield of routine screening remain uncertain. These considerations underscore the need for a more targeted, risk‐based screening approach. In light of the limited data in this specific population, our study aimed to determine the frequency of acromegaly among patients with long‐standing, poorly controlled type 2 DM managed in outpatient settings.

## 2 Materials and Methods

### 2.1 Study Design

This cross‐sectional study included a total of 504 consecutive patients diagnosed with Type 2 DM according to the American Diabetes Association (ADA) criteria [8] with poor glycemic control, who presented at the Endocrinology and Metabolism Outpatient Clinic of Health Sciences University, Dışkapı Yıldırım Beyazıt Training and Research Hospital between 2019 and 2021. The patients were evaluated regarding anthropometric and demographic data, laboratory characteristics, and serum IGF‐1 levels. An evaluation of the data was also made in the previously recorded patients' files.

### 2.2 Patient Selection and Diagnosis

The patients included in the study were those with glycated haemoglobin A1c (HbA1c) of > 8.0% as Type 2 DM with poor glycemic control and those with blood glucose that could not be controlled despite at least 1 year of various treatment regimens (insulin alone or insulin together with oral anti‐diabetic drugs). The serum IGF‐1 levels measured in these patients were evaluated. When a high IGF‐1 level was determined, first the IGF‐1 was measured again, then GH measurement was performed with an oral glucose tolerance test (OGTT). The OGTT was performed after achieving temporary glycemic stabilisation under close clinical observation. In the OGTT, serum GH levels were measured at 0, 30, 60, 90, and 120 min. For patients with high IGF‐1 and insufficient GH suppression or elevated IGF‐1 with an incompatible post‐glucose GH level, the hypophysis was evaluated with magnetic resonance imaging (MRI). The diagnosis of acromegaly was established based on typical clinical features and a serum IGF‐1 level exceeding 1.3 times the upper limit of normal (ULN) for age [9]. For patients with equivocal IGF‐1 results, an OGTT was performed to assess GH suppression.

Patients were excluded from the study if they had a known pituitary disorder, a recent diagnosis of DM, Type 1 DM, pregnancy or any condition linked to reduced GH or IGF‐1 such as kidney or liver disease, uncompensated thyroid dysfunction, malnutrition, or the use of estrogen.

### 2.3 Measurement of GH and IGF‐1

Serum GH and IGF‐1 levels were measured using an IMMULITE 2000 Immunoassay System (Siemens Healthcare GmbH, Erlangen, Germany). The GH measurement method was based on the solid‐phase, two‐region chemiluminescence immunometric test (Siemens Healthcare Diagnostics Products, Glyn Rhonwy Llanberis, Gwynedd, UK). The GH measurement was standardised using the recombinant WHO standard, 2.IS98/574. According to the manufacturer's instructions, the general study internal and total variation coefficients (CV) calculated for GH for the quality control range of 2.6–17 mcg/L are 2.9%–4.6% and 4.2%–6.6%, respectively.

The IGF‐1 assay method was a solid‐phase, enzyme‐labelled chemiluminescence immunometric assay (Siemens Healthcare Diagnostics Products, Glyn Rhonwy Llanberis, Gwynedd, UK). According to the manufacturer's instructions, the general study internal and total variation coefficients (CV) calculated for IGF‐1 for the quality control range of 77–1358 ng/mL are 2.3%–3.9% and 3.7%–7.7%, respectively. The serum IGF‐1 levels were compared with the normal age and gender‐adjusted reference values obtained from the manufacturer's instructions for use.

### 2.4 Statistical Analysis

Data obtained in the study were analysed statistically using JMP 16.0.0 software (SAS Institute, Cary, NC, USA). Conformity of the data to normal distribution was tested with the Kolmogorov–Smirnov and Shapiro–Wilk *W* tests. Results were presented as mean ± standard deviation (SD) or median (interquartile range) values, or number (*n*) and percentage (%). Numerical variables were compared using the Paired Samples *t*‐test or Mann–Whitney *U* test and the chi‐squared test or Fisher's exact test were used for categorical variables. The level of statistical significance was defined as *p* < 0.05.

## 3 Results

Of the 504 patients screened, elevated IGF‐1 levels were observed in two patients (0.39%), but none met the diagnostic criteria for acromegaly. This corresponds to an overall acromegaly prevalence of 0.19% in the study population.

The study cohort included 310 females (61.5%) and 194 males (38.5%) with a mean body mass index (BMI) of 31.23 kg/m^2^. The mean age was 57.69 ± 10.84 years (57.8 years for females, 57.4 years for males). The median duration of diabetes was 9.0 (3.0–15.0) years, and the mean HbA1c was 11.12% ± 2.30%. Comorbidities included hypertension in 248 patients (49.2%), dyslipidemia in 127 (25.1%), and coronary artery disease in 127 (25.1%). The baseline demographic and clinical characteristics, risk factors for atherosclerosis, and diabetic complications are summarised in Table 1.

**TABLE 1:** Clinical characteristics including the risk factors for atherosclerosis and diabetic angiopathies of the patients with T2DM.

Characteristics and risk factors	Ratio or mean ± SD	Characteristics and risk factors	Ratio or mean ± SD
Gender (M/F)	194/310	GH (ng/mL)	1.58 ± 13.64
Age (years)	57.69 ± 10.84	IGF‐1 (ng/mL)	108.85 ± 38.42
T2DM duration (years)	9.75 ± 7.70	Serum LDL cholesterol (mg/dL)	114.88 ± 42.28
Body Mass Index (kg/m^2^)	31.23 ± 12.67	Serum triglycerides (mg/dL)	202.19 ± 138.86
HbA1c (%)	11.12 ± 2.30	Serum AST (U/L)	21.76 ± 20.96
Hypertension, (%)	49	Serum ALT (U/L)	23.59 ± 20.10
Dyslipidemia, (%)	71	Serum platelet (× 103/μL)	271.16 ± 81.29
Coronary arter disease, (%)	25	Serum creatinine (mg/dL)	0.88 ± 0.46
Albuminuria‐spot UACR (mg/g kreatinin)	95.02 ± 266.20		
Diabetic angiopathies			
Retinopathy, (%)	24	Microalbuminuria ≥ 30–299	28
Neuropathy, (%)	56	Macroalbuminuria ≥ 300	6
Nephropathy, (%)	34		

Among the 504 patients with poorly controlled Type 2 DM, none had been previously suspected of acromegaly by their treating physicians. High serum IGF‐1 levels were identified in only two patients (0.39%) relative to age‐ and sex‐adjusted reference ranges (Table 2). The IGF‐1 levels in these two patients were 270 and 230 ng/mL, respectively.

**TABLE 2:** Clinical characteristics of the patients with a high IGF‐1 level.

No	Age (years)	Gender	BMI (kg/m2)	HbA1c (%)	APG (mg/dL)	GH (ng/mL)	IGF‐1 (ng/mL)	1.3 × ULN of IGF‐1 (ng/mL)
1	61	M	29.7	9.1	135	0.7	270 (75–212)	275.6
2	65	F	23.8	15.3	227	1.58	230 (75–212)	275.6

**FIGURE 1:**
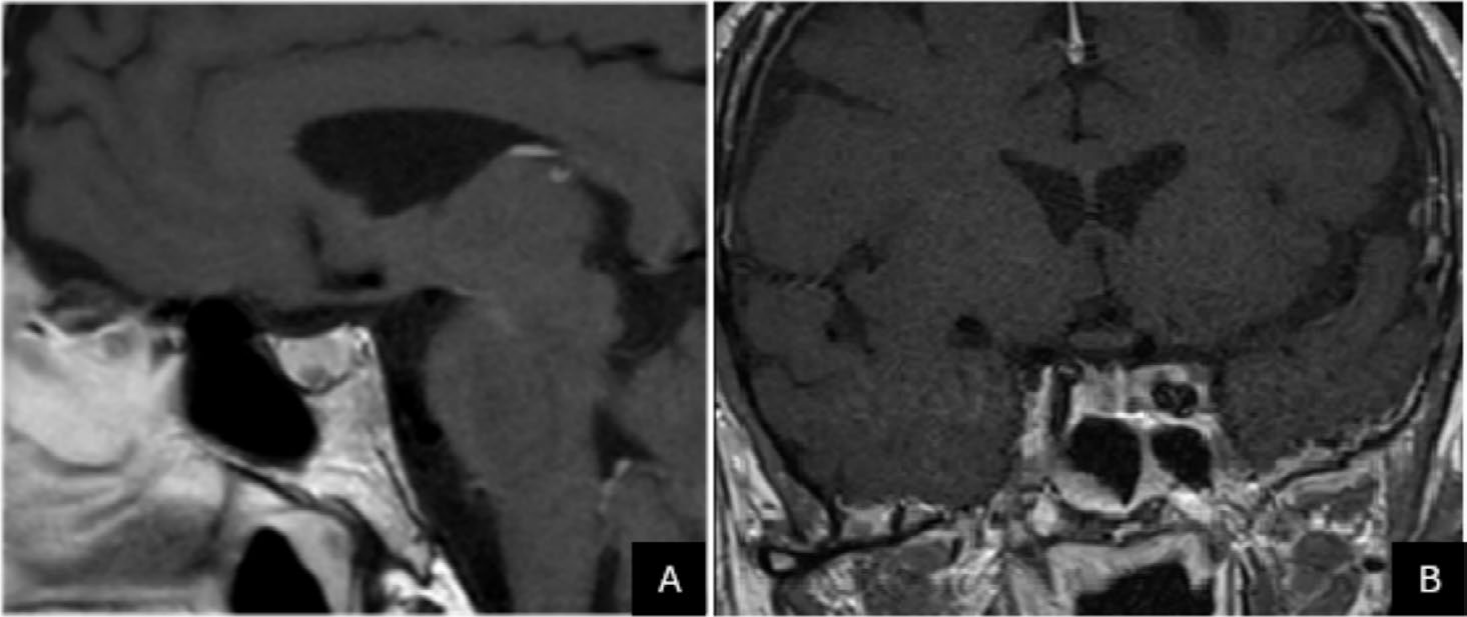
Pituitary MRI of patient revealed a 4 × 3 mm sized millimetric lesion that does not show enhancement in T1AG contrast‐enhanced series (cystic microadenoma with dense content?), sagittal (A), coronal (B).

Both patients underwent an oral glucose tolerance test (OGTT) for GH assessment. In Patient 1, GH levels at 0, 30, 60, 90 and 120 min were 1.5, 0.46, 0.23, 0.14 and 0.12 ng/mL, respectively, demonstrating adequate suppression. In Patient 2, GH levels were 2.26, 2.19, 1.32, 0.96 and 0.88 ng/mL, indicating borderline suppression. Pituitary MRI performed for Patient 2 demonstrated a 4 × 3 mm cystic lesion that may represent a microadenoma (Figure 1). However, this patient exhibited no clinical features of acromegaly, and the GH response during OGTT did not meet the diagnostic threshold. Therefore, acromegaly was not confirmed in either case.

## 4 Discussion

The results of the current study on patients with poorly controlled Type 2 DM revealed that the prevalence of high IGF‐1 was only 0.19%. In this cohort, two patients exhibited elevated IGF‐1 but had no phenotypic features of acromegaly, and GH was adequately suppressed during OGTT, ruling out acromegaly. The detection of this isolated biochemical abnormality without clinical or hormonal evidence of acromegaly indicates that routine screening in all poorly controlled diabetic patients may not be warranted.

IGF‐1 levels can rise in some patients with hyperthyroidism and in pregnant women, though it is rare for levels to be elevated in conditions other than acromegaly [1]. The 2014 Endocrine Society Acromegaly Guidelines recommend measuring IGF‐1 levels in patients exhibiting typical clinical signs of acromegaly, particularly in the presence of classic acral and facial features, or even in the absence of typical symptoms in patients with related conditions such as sleep apnea, hypertension, Type 2 DM, arthritis, carpal tunnel syndrome, and hyperhidrosis. The suggested approach is to measure serum IGF‐1 in patients with a high or uncertain serum IGF‐1 level to confirm the diagnosis by assessing GH values following hyperglycemia identified during an OGTT [5]. To identify cases of acromegaly among patients with diseases linked to it and thereby establish a risk group with specific clinical and somatic characteristics warranting further diagnostic evaluation, Antsiferov et al. conducted a study that involved administering a questionnaire to 1249 patients with various systemic and metabolic disorders. Acromegaly was suspected in 367 (29.4%) of these patients, who then underwent advanced testing. Most of these patients (79.3%) had previously been monitored for DM. Consequently, 9 of the 1249 patients with comorbidities were newly diagnosed with acromegaly [10].

Despite advancements in diagnostic techniques and efforts to promote early detection, there has been no significant reduction in the time from symptom onset to the diagnosis of acromegaly [11]. Such delays contribute to psychosocial deterioration and an increased risk of complications [12]. At the time of diagnosis, approximately 70% of patients present with a microadenoma, likely reflecting the impact of delayed recognition [2]. A significant gap exists in routine acromegaly screening within the general population, and active screening programs could potentially identify numerous undiagnosed cases [3]. In the current study of patients with poorly controlled Type 2 DM, one patient exhibited a small pituitary lesion and borderline IGF‐1 elevation but lacked clinical or biochemical evidence of acromegaly. These findings suggest that routine active screening in this specific population has limited diagnostic yield, and its cost‐effectiveness remains uncertain.

The prevalence of acromegaly appears higher than previously reported, with many patients presenting a milder phenotype [13]. Due to the broad spectrum of symptoms, patients often seek care from various specialties, including dentistry, hand surgery, ophthalmology, and gynaecology. Subtle facial and acral changes are frequently misattributed to natural aging, further contributing to diagnostic delays [14]. These factors underscore the need for innovative strategies to improve diagnostic awareness. Recent studies using artificial intelligence to recognise acromegalic facial features show promising potential for aiding earlier identification [14, 15].

Several studies, similar to the present one, have sought to identify acromegaly among patients with impaired carbohydrate metabolism. Rosario et al. reported acromegaly in 3 of 2270 adults (aged 20–70 years) with Type 2 DM or glucose intolerance, corresponding to a prevalence of approximately 480 cases per million population [6]. Likewise, Suda et al. retrospectively examined hospitalised patients with poorly controlled Type 2 DM and identified 2 cases among 317 patients, noting that the prevalence of acromegaly increased with age [7]. While Rosario et al. neither recommended nor discouraged routine screening, they emphasised that early detection efforts inevitably increase the number of suspected cases requiring further evaluation and highlighted the importance of improving physician awareness of acromegaly. Similarly, Suda et al. suggested that IGF‐1 screening may be useful in hospitalised patients with poorly controlled Type 2 DM. In our outpatient cohort, however, the diagnostic yield was very low, and no active acromegaly was identified. These findings suggest that while selective screening in specific high‐risk groups may hold potential, routine IGF‐1 testing in all outpatients with poorly controlled diabetes may have limited benefit. Larger, controlled studies are warranted to clarify which subgroups would most benefit from targeted screening and to assess the cost‐effectiveness of such strategies.

This study has certain limitations. The relatively small sample size may have limited the ability to identify very rare cases of acromegaly. In addition, the study was conducted in a single tertiary outpatient setting, which may limit the generalizability of the findings to broader diabetic populations. Finally, as a cross‐sectional study, it cannot establish a causal relationship between poor glycemic control and undiagnosed acromegaly.

In conclusion, improving the early and accurate detection of acromegaly requires strategies that enhance diagnostic awareness and facilitate timely identification. Selective screening of patients with suggestive clinical or metabolic features remains a promising approach. In this study, no cases of active acromegaly were identified among outpatients with poorly controlled Type 2 DM, indicating that universal IGF‐1 screening in this population may have limited diagnostic yield. Instead, IGF‐1 measurement may be considered in patients with poor glycemic control who also exhibit phenotypic features or comorbidities suggestive of acromegaly. Further studies are warranted to refine risk‐based screening algorithms and to assess the cost‐effectiveness of such targeted approaches.

## Author Contributions

M.T.S. and M.K. conceptualised and designed the article. M.T.S., H.B., U.G., H.D., and S.H. collected and interpreted the data. M.T.S. wrote the first draft of the article. H.B., B.U., E.C., and M.K. reviewed and supervised the manuscript critically. All authors approved the final version of the manuscript and agree to be accountable for all aspects of the work.

## Funding

The authors have nothing to report.

## Ethics Statement

This study was performed in line with the principles of the Declaration of Helsinki. Approval was granted by the Ethics Committee of the Health Sciences University, Dışkapı Yıldırım Beyazıt Training and Research Hospital (newly Ankara Etlik City Hospital) (Date: 12.07.2021/Number: 115/13).

## Consent

Informed consent was obtained from all individual participants included in the study.

## Conflicts of Interest

The authors declare no conflicts of interest.

## Data Availability

The data that support the findings of this study are available from the corresponding author upon reasonable request.
